# Medication administration errors for older people in long-term residential care

**DOI:** 10.1186/1471-2318-11-82

**Published:** 2011-12-07

**Authors:** Ala Szczepura, Deidre Wild, Sara Nelson

**Affiliations:** 1 Warwick Medical School, University of Warwick, Coventry, UK; 2 Faculty of Health and Social Care, University of the West of England, Bristol, UK

## Abstract

**Background:**

Older people in long-term residential care are at increased risk of medication prescribing and administration errors. The main aim of this study was to measure the incidence of medication administration errors in nursing and residential homes using a barcode medication administration (BCMA) system.

**Methods:**

A prospective study was conducted in 13 care homes (9 residential and 4 nursing). Data on all medication administrations for a cohort of 345 older residents were recorded in real-time using a disguised observation technique. Every attempt by social care and nursing staff to administer medication over a 3-month observation period was analysed using BCMA records to determine the incidence and types of potential medication administration errors (MAEs) and whether errors were averted. Error classifications included attempts to administer medication at the wrong time, to the wrong person or discontinued medication. Further analysis compared data for residential and nursing homes. In addition, staff were surveyed prior to BCMA system implementation to assess their awareness of administration errors.

**Results:**

A total of 188,249 medication administration attempts were analysed using BCMA data. Typically each resident was receiving nine different drugs and was exposed to 206 medication administration episodes every month. During the observation period, 2,289 potential MAEs were recorded for the 345 residents; 90% of residents were exposed to at least one error. The most common (n = 1,021, 45% of errors) was attempting to give medication at the wrong time. Over the 3-month observation period, half (52%) of residents were exposed to a serious error such as attempting to give medication to the wrong resident. Error incidence rates were 1.43 as high (95% CI 1.32-1.56 p < 0.001) in nursing homes as in residential homes. The level of non-compliance with system alerts was very low in both settings (0.075% of administrations). The pre-study survey revealed that only 12/41 staff administering drugs reported they were aware of potential administration errors in their care home.

**Conclusions:**

The incidence of medication administration errors is high in long-term residential care. A barcode medication administration system can capture medication administration errors and prevent these from occurring.

## Background

The care home sector is an increasingly important provider of long-term care for older people. A review of the international literature has recently identified that research in the area of quality and safety is lacking, especially for residential homes which have no on-site nursing staff [1]. A number of authors have identified prescribing and management of medication more generally as key areas for improved patient safety in care homes [2-9]. In England, over 18,000 homes currently provide beds for more than 453,000 people, compared to 167,000 beds in hospitals. The majority of residents are older people with complex health needs. Six out of ten are cared for in a residential home with no on-site nurses. In such homes the management of prescribed medication is undertaken by non-nursing, social care staff who may have had no formal training in safe practice [10]. In nursing homes, which must have a registered nurse (RN) on site 24 hours per day to meet regulation requirements, medicine administration is one of the many tasks carried out by busy RNs. In both settings, prescribing decisions are the responsibility of the general practitioner (GP) or the hospital physician.

It is known that in England 45% of all care homes in 2005 failed to meet the minimum standard for medication management [4], and that this figure remains high at 28% in 2010 [11]. A cross-sectional study of a sample of 256 residents in 55 UK care homes found that 69.5% had been exposed to one or more medication errors [12]; these included mistakes made by GPs in prescribing, dispensing errors by pharmacies, and administration errors made by care home staff.

To guard against drug administration errors in hospital care settings, electronic medication administration recording (eMAR) has been widely implemented to replace paper-based systems [13]. eMAR systems have now been developed for use in long-term residential care environments. It is reported that safety is now being further improved in hospitals by the use of barcode technology integrated with eMAR systems [14]. Similar systems are being developed for use in long-term residential care.

We report on a study of the first barcode medication management system specifically developed for use in UK residential and nursing homes, with external pharmacy-led data capture, processing and record management. The main aim of the research was to examine the incidence of potential medication administration errors (MAEs) in nursing and residential homes using the barcode medication administration (BCMA) system. A further objective was to compare observed error rates and response to system-alerts for residential and nursing homes. Other aspects of the system such as bar-coded dispensing, clinical readings, and stock management were not considered in the present study.

## Methods

### Study overview

The Proactive Care System (PCS) (See Additional file 1) was introduced into a cross-section of nursing and residential homes. The study collected real-time, longitudinal data on all attempts to administer medication to residents using a disguised observation technique [15]. Following a 4 week period to allow staff to familiarise themselves with the new technology (learning curve), anonymised data were collected over the 3-month period. Staff, resident and medication details were recorded for every administration attempt. A health technology assessment estimated effectiveness in terms of the number of potential errors averted [16]. The definition adopted for medication administration errors was "any deviation between the medication as prescribed and that administered" [12].

### Setting

The study was undertaken in 13 care homes (9 residential and 4 nursing) representing a geographical spread covering the South West, Midlands and North West of England. Study sites included small and large independent care providers from both commercial and not for profit sectors. All homes were rated as being of a good or higher standard by national regulator inspection. Staff who administered medication were trained in use of the new technology.

All residents in receipt of medication throughout the study period were included in the patient cohort. A convenience sample of 45 staff responsible for management and administration of medication was invited to complete the pre-study questionnaire.

### Ethics approval

The study obtained ethics approval from the University of the West of England Ethics Committee HSC (Health and Social Care) on 25th July 2008 Ref HSC/08/07/47. All participants received project written information prior to request for written consent. All participants' questionnaires were anonymous; participants were identifiable by codes known only to the researcher (SN). No resident was capable of identification in computer-related data files.

### Barcode medication administration (BCMA) system

The pharmacy-managed, barcode medication administration system differs from a simple eMAR system in its design and functionality (See Additional file 1). All data management is undertaken centrally by the pharmacy outside the care home setting. At the end of each week a report is sent to the care home manager with details of all potential administration errors and the member of staff involved. During a medication round, the user first scans each patient's barcode identifier using a hand-held device to ensure the correct drug file is recalled and to visually confirm identification of the resident. The user then scans each dispensed item prior to administration. The system carries out a number of checks based on both bar codes to ensure the following are correct (i) resident, (ii) medication, (iii) time, (iv) dose, (v) quantity and (vi) in date. If administration is outside any parameter, the system alerts the member of staff immediately to the potential error. If administration of a medicine within the correct time window lapses the system enters this as a 'missing record'. The system records all deviations between the medication as prescribed and that finally administered.

### Data collection and analysis

#### Medication administrations

Anonymised data on every barcode medication administration taking place 24 hours per day over a 3-month period were extracted from the central data system. Data were downloaded as Excel files and subsequently transferred for analysis into the SPSS statistical package. Descriptive analysis provided information on the numbers of residents receiving medication, the number of medications per resident, and the number of administrations given (with or without scanning of barcodes). The level of compliance was estimated in terms of the number of deviations from medication as prescribed (see above) versus the total number of administration episodes. Where the central system recorded the same type of error repeatedly within a short time period for an administration (i.e. continued attempts to administer medication incorrectly after initial alert), this was counted as a single potential error and only final compliance with these alerts was included.

#### Pattern of medication administration errors

The types and incidence rates for potential MAEs were examined. Potential MAEs were placed in broad thematic categories related to: incorrect timing; attempts to give medication to the wrong person; and administration of medication that had been discontinued. If the data indicated that a user alert was triggered by an attempt to administer medication slightly early (i.e. within 10 minutes of the prescribed time) this was excluded since it was judged likely to have only minor consequences. Error incidence rates (based on the total number of medication administrations) were estimated globally and separately for the two types of long-term care settings. Relative rates were compared for residential and nursing homes and a 95% Confidence Interval (CI) calculated based on incidence rate ratios using STATA 1C 10. More detailed analysis of individual records, to identify patterns which might offer an explanation for the occurrence of a particular type of error, was also undertaken.

#### Pre-study staff views on medication administration errors in their care home

A pre-study questionnaire (see Additional file 2) collected staff views, based on their experience of using paper-based medication administration recording (MAR) charts, on the following: (i) awareness of 'near misses' in their home i.e. times where an error had almost occurred but the administrator had noticed just in time (Q15); (ii) which, if any, in a list of common errors they had observed in their home (Q6); (iii) what are perceived to be the most common reasons for drug administration errors (Q5); and (iv) their level of confidence (Likert scale) when undertaking medicine rounds as sole administrator (Q17). The questionnaire was piloted in a care home that was not part of the study. Responses were analysed globally and separately for residential home staff and RNs in nursing homes. Levels of self-confidence in undertaking medicine rounds were compared using the Man-Whitney *U *test.

## Results

### Medication administrations

A total of 345 residents in the 13 care homes were receiving medication throughout the study period; 245 in residential homes and 91 in the nursing homes. Typically, staff were administering nine different drugs to patients. The number was similar in the two settings; 9.0 medications per resident in the nursing homes and 8.8 in residential homes.

During the study period, residents were exposed to a total of 213,220 separate medication administration episodes, equivalent to 206 per resident each month.

### Pattern of medication administration errors

The barcode hand-held device provided data on 88% (n = 188,249) of administrations. These formed the basis of the analysis of potential administration errors. Possible reasons for absence of barcode scanning data for the remainder are described in Additional file 1.

#### (i) Frequency of potential medication administration errors

A total of 2,289 potential MAEs were identified over the study period. This represented 1.2% of barcode medication administration episodes. Table 1 provides a breakdown of these. Failure to comply with system alerts was extremely infrequent. Over the study period, there were a total of only 142 such occasions (75 in the nursing homes and 67 in the residential homes). This equates to 0.075% of all barcode administrations.

**Table 1:** Number of potential medication administration errors (MAEs) in 3-month observation period

	All Homes (345 residents)	Residential Homes (254 residents)	Nursing Homes (91 residents)
Total barcode medication administration episodes	188,249	136,340	51,909

Total number averted MAEs	2,289	1,481	808

Frequency averted MAEs	1.22%	1.09%	1.56%

Mean number barcode administrations per resident	545.6	536.8	570.4

Mean number averted MAEs per resident	6.6	5.8	8.9

**Figure 1:**
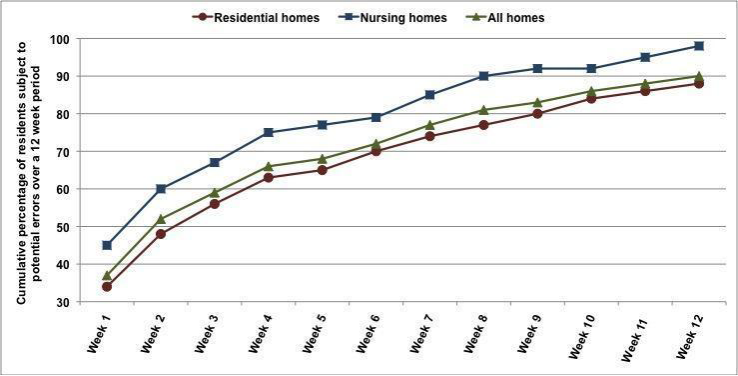
**Percentage of residents exposed to a potential medication administration error (MAE) during 3-month observation period**.

In any one week, the percentage of residents for whom the risk of a medication administration error was averted ranged from 30% to 39%. Figure 1 indicates that, over the 3-month observation period, 90% of residents were exposed to at least one potential MAE. Thus, medication administration errors were not concentrated in a few residents. Risk exposure was higher for residents in nursing homes (98%) than for those in residential care (88%). On average residents were exposed to 6.6 potential MAEs in the observation period.

#### (ii) Types of error averted

Table 2 presents medication administration errors broken down by type of error. The overall incidence rate ratio for nursing and residential homes was 1.43 (95% CI 1.32 to 1.56 p < 0.001). Errors were between 1.32 and 1.56 times as likely to occur in the nursing homes as in the residential homes.

**Table 2:** Types of potential medication administration errors (MAEs) in 3-month observation period

Type of potential MAE	All Homes No. (%)^1^	Residential Homes No. (%)^1^	Nursing Homes No. (%)^1^	Incidence Rate Ratio^2^ (95% CI)
Attempting to give a 4-hourly medication too early (< 3.50 hrs)	1,021 (0.5)	604 (0.4)	417 (0.8)	1.81 (1.60 - 2.05)**

Attempting to give other medication at wrong time	586 (0.3)	412 (0.3)	174 (0.3)	1.11 (0.93 - 1.32)

Attempting to give medication on the wrong day	359 (0.2)	231 (0.2)	128 (0.2)	1.46 (1.17 - 1.80)**

Attempting to give medication to the wrong resident	233(0.1)	164 (0.1)	69 (0.1)	1.11 (0.83 - 1.46)

Attempting to give a medication that has been discontinued	90 (0.05)	70 (0.1)	20 (0.04)	0.75 (0.46 - 1.23)

**Overall Total**	**2,289 (1.2)**	**1,481(1.1)**	**808 (1.6)**	**1.43** (1.32 to 1.56)**

**^1 ^**Error incidence rate calculated as % of total administrations in 3 month observation period

**^2 ^**Ratio of rate in nursing homes compared to residential homes. 95% confidence interval (CI) calculated for incidence rate ratio.

** Significant *p *< 0.001

Table 2 shows that the most frequently recorded error was attempting to give a 4-hourly medication too early. One half of administration errors fell into this category. When individual records were examined in more depth they showed attempts to give medications such as paracetamol prematurely which had been given in the last 4 hours.

The second most frequently error was attempting to give other medications later or earlier than the prescribed time. A quarter of all averted administration errors fell into this category. More detailed examination of these records suggested that staff, who have set times for medication rounds, were trying to fit as many medication administrations as possible in a round rather than following prescribed times. An associated, potentially more serious error-type was attempting to give medication on the wrong day. Closer analysis of these records indicated that such errors were often linked to one day's dose having been given and a different administrator, who was unaware of this, trying to give the same dose again in the same 24 hour period. Examples included agency staff unfamiliar with the resident and their regime.

The final two error-types listed in Table 2 are potentially the most serious. The first, which involved staff attempting to give medication to the wrong resident, represented one in ten near misses in both settings. The second, attempting to give a medication that had been discontinued, represented one in twenty five potential MAEs. Over the 3-month observation period, 52% of residents were exposed to one or both of these more serious errors.

Comparison of different care settings shows that attempts to give a 4-hourly medication too early were 1.81 times as likely to occur in nursing homes as in residential homes (95% CI 1.60 - 2.05 p < 0.001). Similarly, attempts to give other medication later or earlier than prescribed were 1.46 times more likely in nursing homes (95% CI 1.17 - 1.80 p < 0.001). However, there were no significant differences between residential and nursing homes in the incidence of more serious errors such as attempts to give medication to the wrong person.

### Pre-study staff views on medication administration errors in their care home

All 45 staff who were invited to fill in the pre-study questionnaire completed the survey. These included 31 staff from the residential homes (7 home managers and 24 social care staff); and 14 nursing home staff (3 RN managers and 11 RNs). In nursing homes, all the RNs held a level 1 registered nurse qualification. In the residential homes, 5 non-nursing, social care staff held the National Vocational Qualification (NVQ) at level 4, 18 staff held NVQ at level 3, and 7 staff the basic social care level 2 qualification [17].

#### (i) General awareness of 'near miss' medication administration errors

Table 3 shows that before technology introduction nearly one third of staff overall reported that they were aware of potential medication administration errors being averted in their care home, commonly referred to as 'near misses'. However, although social care staff responded positively to this question, no RNs reported a similar awareness of near misses in their nursing home.

**Table 3:** Pre-study staff awareness of potential medication administration errors being averted in their care home ('near misses')

Response	All Homes No. staff (%)	Residential Homes No. Care staff (%)	Nursing Home No. RN staff (%)
Yes	12 (29)	12 (41)	0 (0)

No	29 (71)	17 (59)	12 (100)

**Total**	**41^1 ^(100)**	**29^1 ^(100)**	**12^1 ^(100)**

**^1 ^**Four missing values, two in each setting

#### (ii) Common types of administration error observed with current paper-based system

Based on their experience of paper-based MAR charts, staff were most likely to consider that 'missed medication' was a common error (see Table 4). Fewer than half (44%) of staff in both types of home agreed that 'medication was given at the wrong time'. Only residential home staff reported being aware of more serious errors such as 'medication given to the wrong resident' (44%) or the 'wrong medication given' (29%). However, both staff groups agreed they had observed errors such as 'wrong dosage given' and 'discontinued medication given', although a higher percentage of social care staff than RNs agreed with these statements.

**Table 4:** Medication administration errors with paper-based MAR systems

	Staff agreeing they have observed this type of error		
**Type of error**	**All Homes** **(N = 45 staff)** **No. Staff (%)**	**Residential Homes** **(N = 31 staff)** **No. Care staff (%)**	**Nursing** **Homes** **(N = 14 staff)** **No. RN staff (%)**

Medication missed	31 (69)	23 (74)	8 (57)

Medication given at wrong time	20 (44)	14 (45)	6 (43)

Medication given to wrong person	15 (33)	15 (48)	0 (0)

Wrong medication given	13 (29)	13 (42)	0 (0)

Wrong dosage given	12 (27)	10 (32)	2 (14)

Discontinued medication given	8 (18)	7 (23)	1 (7)

#### (iii) Most common reason contributing to administration errors

Nearly all staff identified 'interruptions during round' as a contributory cause for administration errors (Table 5). Around half also agreed that being 'stressed' or 'under pressure to complete the round' contributed, with the first more frequently identified in residential homes and the second in nursing homes. No respondent identified 'lack of training'. RNs also did not agree with reasons such as 'insufficient knowledge of medication' or 'present system confusing and open to error', although a small number of social care staff did. Responses indicated that both staff groups consider administration errors are caused by a combination of distractions and work pressures.

**Table 5:** Staff agreement with postulated reasons for medication errors

	Staff agreeing with reason stated		
**Reason for error**	**All Homes** **(N = 45 staff)** **No. Staff (%)**	**Residential Homes** **(N = 31 staff)** **No. Care staff (%)**	**Nursing Homes** **(N = 14 staff)** **No. RN staff (%)**

Interruptions during round	43 (96)	31 (100)	12 (86)

Staff stressed	23 (51)	20 (65)	3 (21)

Under pressure to complete round	21 (47)	12 (39)	9 (64)

Shortage of staff	6 (13)	5 (16)	1 (7)

Current system confusing and open to error	4 (9)	4 (13)	0 (0)

Insufficient knowledge of medication	2 (4)	2 (7)	0 (0)

Lack of training	0 (0)	0 (0)	0 (0)

#### (iv) Level of confidence when undertaking medicine rounds alone

Staff responsible for administering medication appeared to be at ease with carrying out medicine rounds on their own. Based on a Likert scale of 1-7 (1 = *not at all at ease*, and 7 = *extremely at ease*), there was no significant difference between staff in residential homes (mean score 6.0) and nursing homes (mean score 6.5) in this respect (Man-Whitney *U *test, p > 0.05). There was also no significant association between mean scores for level of confidence and the qualifications a member of staff had achieved; RNs (mean score = 6.5), NVQ4 (7.0), NVQ3 (5.9) and NVQ2 (6.1).

## Discussion

Medication management covers the whole process from prescribing, through to dispensing and finally administration of medicines. Errors in any one of these steps can have serious consequences for the patient. Although such errors are acknowledged to be preventable [10], currently they still result in considerable morbidity, mortality and healthcare utilisation by older people [2-6]. According to the United States (US) Food and Drug Administration, over 770,000 patients are injured annually because of medication errors [18]. Administration errors account for 38% of these events. In the US, it is reported that up to 35% of older people in the community may experience some form of adverse medication event each year [19]. The incidence is thought to be even higher among nursing home residents [20]. In Italy, up to 30% of hospital admissions in older people are related to such events [21]. In the UK, 9% of hospital admissions for people aged 60 and over are as a result of 'poisonings by drugs, medicaments and bio substances' [22]. In 2005, 76,692 admissions to English hospitals were associated with an adverse drug reaction; this number increased by 45% over the period 1998 to 2005 and 59% of all cases involved patients aged over 60 years [23].

Older people are at increased risk of medication-related adverse events due to a combination of factors including multiple medication (polypharmacy) and age-related changes in the body's response to medicines [24]. Polypharmacy is extremely common in care homes, with residents generally reported to receive seven or more items each [25]. Residents in the current study received an average of nine different drugs. In such a situation, the risk of incorrect administration of a prescribed medication is high, with the potential to result in a large number of adverse events [26]. To date, most studies of improving medication safety in care homes have focused on prescribing [27]. Relatively little research has examined administration of prescribed medicines and how the safety of this might be improved. A comprehensive literature review has drawn attention to a general lack of evidence on this aspect of safety and quality improvement in care homes, in particular residential homes [1]. The current research provides the first incidence figures for medication administration errors in UK residential, as well as nursing, homes.

With the number of people aged 75 and over in the UK projected to nearly double by 2033, increasing from 4.8 to 8.7 million [28], the quality of clinical care provided to older people will increasingly affect national patient safety. The care sector in the UK relies heavily on residential homes which have no on-site nursing and, as older people's clinical needs increase, innovative ways of providing clinical care and increasing the expertise of non-nursing, social care staff will be required [29]. New technology may have a part to play in this, especially for aspects such as medication. Historically, quality improvement interventions for preventing medication errors have included labour intensive manual medication reviews, inspection of prescription requests and authorised prescriptions, stock checks, inspection of dispensed items and audit of medication administration charts [30]. More recently, systematic reviews of the literature have provided evidence that computerised support systems can improve prescribing and dispensing practices, but there is limited evidence of their impact on administration of medication for older people [31,32].

The present study is the first to assess the introduction of a barcode medication administration system in UK long-term residential care. In terms of the level and pattern of errors we observed, research from the US has found a similarly high level of medication administration errors, with 'wrong time' the most frequently observed error in hospitals, skilled nursing facilities and assisted living environments [33-35]. Our findings indicate that, over a three month observation period, 90% of residents were exposed to at least one potential administration error. This figure mirrors that reported in an earlier study of care homes in England although, because a smaller number of administrations was examined in this study and sampling methods differed (See Additional file 3), rates cannot be directly compared [12]. In the present study, overall error rates were higher in nursing homes, where RNs undertake medication rounds.

The pre-study survey suggests that errors are linked to system and behaviour factors rather than a lack of education or training. Staff in both settings identified interruptions to medicine rounds as the major cause of errors, supported by several other authors [36-43]. Neither staff group associated errors in medication administration with lack of training. The fact that recorded levels of confidence were not linked to qualification levels would seem to support this. Instead, it appears that the concentration necessary for safe administration of medicines is interrupted by competing demands upon staff time. Our pre-study survey also indicated that RNs in nursing homes appeared to be generally less aware of the potential for errors to occur in administration than their residential home social care staff counterparts.

Some explanation for the higher incidence of medication administration errors observed in nursing homes may lie firstly in the greater complexity of decision-making underpinning the process of administering medication for RNs as opposed to social care staff [44-46]. Since nursing home patients are generally more seriously ill and therefore may have *more complex medication regimes*, this will inevitably raise pressure on staff and increase the risk of administration errors by nurses [47]. In other studies, it has been reported that higher grade nurses are generally more prone to making medication errors than those of a lower grade [48]. Secondly, RNs with their higher level responsibilities are also more likely to have to *multitask when undertaking medication rounds*, further increasing the risk of error [49]. Researchers have recently reported 4.8 ± 6.6 interruptions per medication round for nurses in long-term care facilities [50]. Studies on hospital wards have also shown that the more frequent the interruptions the greater the number of errors [51]. Thirdly, RNs may *employ critical thinking *and clinical judgement, using their knowledge of the patient to make decisions regarding the timing of medication which counter what is prescribed by the physician [45].

The higher incidence of errors recorded for RNs in the study contrasts with their lower pre-study recall of previous near misses. This may be linked to the fact that RNs have a tendency to focus on *'reportable' errors *that have actually occurred more so than ones that have been averted [52], and therefore may not acknowledge the latter. Other research evidence also suggests that conduct of routine, time-consuming tasks such as repeat medication rounds can lead nurses into *complacency *and a diminished sensitivity towards the *potential for harm *resulting from medication errors [53,54]. Medication rounds occupy approximately one-third of nursing time in long-term residential care [50]. In contrast, for social care staff who do not have a robust professional and educational framework or clinical training to support them [55], the sense of being 'stressed' when administering medication could reduce any complacency and increase recall of near misses.

Although there is some research to demonstrate that nursing staff adopt unsafe work-around practices with electronic medication administration systems [56], interestingly there was no evidence of this in the present study. At the same time, there was a high level of acceptability for the system among nursing and social care staff. The final level of non-compliance with medication administration as prescribed was very low in both settings (0.075% of administrations). Presumably, this is because the PCS system by its very nature is extremely difficult to circumvent, with all data management undertaken outside the care home setting and feedback provided on every alert to the care home manager. Compliance may also be linked to the facts that the system was implemented in an institutional setting and that it provided automatic system-initiated alerts. A recent review of the evidence on computerised prescribing decision-support systems concludes that these perform better in institutional rather than ambulatory settings, and when decision support is initiated automatically by the system as opposed to user initiation [57]. In a context in which communication between shifts is imperfect, or there is a high level of agency use, a system with built-in safeguards may also be expected to be more effective.

The main limitations associated with the present study include the relatively small number of care homes studied and the disparity in numbers of nursing and social care staff, and the absence of agreed criteria for valuing the different types of medication administration error observed. In terms of the latter, although a number of approaches have been attempted to categorising medication errors for older people in hospital, community and general practice [58-60], it is only very recently that this debate has extended to care homes [61]. Furthermore, existing criteria concentrate almost exclusively on identifying errors in prescribing, and only rarely errors in administration [62]. There is therefore no consensus currently on the relative importance of different types of administration errors in care homes. Even so, certain errors (such as attempting to give medication to the wrong resident or attempting to give medication that had been discontinued) can clearly be considered as more serious. A further limitation is the assumption that the introduction of the system did not alter behaviour and make staff more careful. However, any Hawthorne effect was minimised by the disguised observational technique used.

Finally, our findings from residential care homes suggest that social care staff in nursing homes might also be trained to administer basic medication using the barcode medication administration system. This would leave registered nurses free to focus on more complex medication regimes and free up valuable nurse time for other tasks in the care home. More research is required into the decision-making of nurses during medication rounds before delegation to care staff in a nursing home setting can be recommended. The potential also exists for data from the system to be used to assess other aspects of medication management, such as prescribing, and provide a low cost decision-support system. A preliminary analysis of PCS prescribing data on antipsychotics and comparison with national guidelines has already demonstrated various short-comings [63]. Research is currently underway to examine prescribing patterns for a range of further medications and to bench-mark these across care homes and GP practices.

## Conclusions

We have found that medication administration errors, such as attempts to give drugs at the wrong time, administer medication to the wrong person or give discontinued drugs, are a serious safety issue for older people in long-term residential care. Few residents avoided exposure to such events, and the consequences of any error will be greater due to the frail elderly population in these institutions. The barcode medication administration system tailored for use in care homes can capture and avert such errors. The system has high acceptability and little evidence of staff adopting unsafe work-around practices.

## List of abbreviations

BCMA system: (Barcode medication administration system); eMAR: (Electronic Medication Administration Recording); GP: (General Practitioner); MAEs: (Medication Administration Errors); MAR: (Medication Administration Record); NVQ: (National Vocational Qualification); PCS: (Proactive Care System); PIN: (Personal Identification Number); RN: (Registered Nurse); US: (United States).

## Competing interests

The authors declare that they have no competing interests. DW and SN received support through an educational grant given to the University of the West of England by Pharmacy Plus Ltd for the study. The study design, methods and materials were prepared by an independent academic team (the authors) with IPR retained by the lead University.

## Authors' contributions

DW, AS and SN conceived the idea for the study. SN obtained the data, undertook the surveys and completed the statistical analysis. AS and DW wrote the manuscript which was reviewed by all authors. All authors had full access to all data in the study and can take responsibility for the integrity of the data and the accuracy of the data analysis. All authors read and approved the final manuscript.

## Pre-publication history

The pre-publication history for this paper can be accessed here:

http://www.biomedcentral.com/1471-2318/11/82/prepub

## Supplementary Material

Additional file 1 **Comparison of characteristics of eMAR and pharmacy-managed, barcode medication management systems**. This file provides a more comprehensive description of: • eMAR (Electronic Medication Administration Recording) systems and. • Proactive Care System using pharmacy-managed, barcode medication management. Click here for file

Additional file 2 **The Pro-active Care System and Medicines Management in Care Homes: An Exploratory Study of its Impact: Pre-Introduction Questionnaire**. This file contains the questionnaire used prior to introduction of the technology. Questions covered a large number of areas, including: • demographics, job role, qualifications, work experience; • experiences of medication supply, administration and storage. • personal use of computers in the home and at work and mobile phone use; • pre-PCS introduction SWOT analysis of current system of medication ordering, supply, storage, administration; • sources of job pressure. Click here for file

Additional file 3 **Comparison of current research and Barber *et al*. study (2009)**. This file provides a comparison of the present research and the only other large scale study of medication administration errors in UK care homes, including details of: • study sites. • site selection process. • resident sample studied. • medication administrations observed. • administration errors recorded. Click here for file
