# Supplementary material for: Medication administration errors for older people in long-term residential care
Source: BMC Geriatr. 2011 Dec 7;11:82. doi: 10.1186/1471-2318-11-82 (PMC3254134; doi:10.1186/1471-2318-11-82)
Supplement: Additional file 1 — Comparison of characteristics of eMAR and pharmacy-managed, barcode medication management systems. This file provides a more comprehensive description of: • eMAR (Electronic Medication Administration Recording) systems and. • Proactive Care System using pharmacy-managed, barcode medication management. [file 1471-2318-11-82-S1.DOC]

# Additional files

### Additional file 1 – Comparison of characteristics of eMAR and pharmacy-managed, barcode medication management systems

**1.1 Electronic Medication Administration Record (eMAR) systems**

Over the last decade different parts of the world have seen the introduction of eMAR (Electronic Medication Administration Recording) systems to help provide safety checks and stock management in hospitals and, increasingly, in long term care facilities.

An eMAR system allows an electronic version of the patient’s Medication Administration Record (MAR) chart to be displayed on the computer screen which is usually attached to a drugs trolley. The records are updated via a web interface by the nurse/ carer or a designated person such as a pharmacist.

Whilst there are benefits in moving from a paper based system to an electronic solution in the recording of information, the process of ensuring that the correct patient receives the correct drug remains entirely manual. An eMAR system can usually identify the correct patient and bring the correct record up on the screen. However, the ability to validate the drug being given with the correct one on the screen can only occur if the eMAR system has the ability to recognise the packet concerned. Usually, the drugs being administered either have no barcodes or do not have sufficient information on their barcodes to perform an absolute check. In this respect, a basic eMAR system continues to carry the risk of giving a patient the wrong drug.

**1.2 Pharmacy-managed, barcode medication management systems**

A comprehensive barcode medication management system is designed for administering medication, stock management, clinical readings and communication. In this approach, the pharmacy manages the data on the system which includes information on the patient, their drugs, doses, timings and other prescribed information. The Proactive Care System (PCS) evaluated in this study was developed by Pharmacy Plus Ltd specifically for use in care homes. In this system information is presented on a hand-held device which is synchronised with the pharmacy’s data in real-time. The functionality uniquely allows the system to reconcile the drug being administered with the prescribing information held on file that provides an absolute check at the point of administration.

The core function is delivered with the use of specific barcode identifiers:

1. The “**Patient Barcode**” is an identifier unique to the patient. This is provided by the pharmacy and printed onto a label. It is recommended the label is attached in close proximity to the patient (e.g. their bedroom door, or medication cupboard). However, depending on the procedures within the care home the Patient Barcode could be on the drugs trolley, although this is not ideal.
2. The **Dispensing Barcode** is an identifier unique to the dispensed item. It is printed on every dispensing label from the pharmacy and holds the entire transaction details. The Dispensing Barcode identifies the patient, drug, dose, date, quantity and can link the dispensing transaction back to the prescription from which that medication was dispensed.

In terms of process, care home staff log onto the administration system using a Personal Identification Number (PIN) code. During the medication round, the user scans the Patient Barcode using the device which then shows a picture of the resident to give initial visual confirmation. On confirmation of correct patient, the system computes the drugs that need to be given for this patient and displays this on screen in red. The user is required to read the items on the screen and find the physical medicine in the usual way. However on finding the medicine, the user must now scan the Dispensing Barcode. The system then performs a number of checks to ensure the medicine selected is for: (i) the correct resident; (ii) the correct medication; (iii) the correct time; (iv) the correct dose; (v) the correct quantity; and (vi) in date. If administration is outside any of these parameters, the system alerts the administrator by both displaying a red warning screen with details of the error message and sounding an alarm simultaneously. A successful scan will allow the user to proceed to prepare and administer the medication. The system has also been designed, after initial site testing, to mimic exactly what members of staff do in real practice, thus reducing the likelihood of work-around or non-adherence to the system by busy staff.

The system does allow the user to perform the key processes without barcode scanning. For example, instead of scanning the Patient Barcode, the user can simply select them from a list (but still retain visual confirmation). Also the user can select the drug by reading it from the screen and confirm they have the correct one. In this way, Non-Barcode functionality has been allowed to accommodate those occasions where the medicines are not supplied by Pharmacy Plus or where the barcode was unreadable. However our research showed that whilst allowing for this, the occasions were limited as 88% barcoding was achieved on average. Furthermore, the percentage barcoding by a user is a performance indicator presented to management within the reports.

Because the administration system has the ability to validate the medicine being selected against the prescribed information held on the electronic file the system can capture all events which fail to meet the appropriate criteria and generate a ‘near miss’ event record. This registers the member of staff, the resident, the medication and the error itself. A summary of each near miss is provided to the care home manager for future action. In this respect, the Proactive Care System differs significantly in its design and functionality from other technologies such as eMAR charts.
