# Supplementary material for: Medication administration errors for older people in long-term residential care
Source: BMC Geriatr. 2011 Dec 7;11:82. doi: 10.1186/1471-2318-11-82 (PMC3254134; doi:10.1186/1471-2318-11-82)
Supplement: Additional file 2 — The Pro-active Care System and Medicines Management in Care Homes: An Exploratory Study of its Impact: Pre-Introduction Questionnaire. This file contains the questionnaire used prior to introduction of the technology. Questions covered a large number of areas, including: • demographics, job role, qualifications, work experience; • experiences of medication supply, administration and storage. • personal use of computers in the home and at work and mobile phone use; • pre-PCS introduction SWOT analysis of current system of medication ordering, supply, storage, administration; • sources of job pressure. [file 1471-2318-11-82-S2.DOC]

# Additional files

**Additional file 2 – The Pro-active Care System and Medicines Management in Care Homes: An Exploratory Study of its Impact: Pre-Introduction Questionnaire**

**Date:**

**Participant Code:           Home Code:**

**Age:           Gender:** Male  Female

**Job Role:**

Care Home Manager  Care Worker

Senior RGN  Senior Care Worker

Other RGN  Other (please state) _________________________________

**Grade:**

**Qualifications:**

**RGN Level 1**  **NVQ 2**  **NVQ 3**  **NVQ 4**  **No Qualification**

**Other (please specify)** **__________________________________________________**

**Are you currently in training for NVQ3? Yes**  **No**

**Job title: ________________________________________________________________________________**

**How long working in residential care (months):________________________________________**

**How long working in nursing home care (months)______________________________________**

**Are you: (a) Full-time (b) Part-time**

**If part-time, how many hours/days per week do you work?_______________________________**

**Are you: employed by: The home**  **Nurse Bank**  **Agency**

**The following questions will ask you about your experiences of medication supply, administration and storage. All of your answers are completely confidential and only the research team will see individual questionnaires. You will be identified only by a study code.**

**1. Where are residents’ medications dispensed from?**

(a) A trolley that is taken directly to the residents

(b) A locked cupboard within the resident’s own room

(c) The nurse’s office/treatment room (i.e. trolley stays in treatment room and medication is taken out to the resident by nurse/carer)

(d) Other  (please state)________________________________________________

(e) Any combination of (a), (b), (c) and (d) please state_______________________________

**2. Do you have a recognised medication policy in the home?** Yes  No Not sure

**3. If yes, how often are you required to read this policy?**

Only when starting at the home  Every 6 months  Yearly  No specified time period

Other  (please state)________________________________

(Explore at interview: *How does your policy say you should administer meds, do you comply with this policy? How about if you were busy and under pressure? Is there anything to detect or prevent people from not complying? Have you come across anyone who does not do it this way*)?

**4. Where in the home is this policy kept?_____________________________________________**

**5. Which do you think are the most common reasons for drug errors? (Please tick any that apply).**

Staff are overworked

Staff are under stress

Poor/insufficient knowledge of the action of medications and their side effects

Under pressure to complete drug round in a certain amount of time

Interruptions to the round from other staff and residents

Current system of drug administration is confusing and open to error

Lack of training

Shortage of appropriately qualified staff

Other (please state) ___________________________________________________

**6. Which, if any, of the following errors have you seen in your home? Please tick any that apply.**

Wrong dosage being given  Medication given to the wrong resident

Wrong medication given  Medication given at the wrong time

Medication missed altogether  I have not seen any of these errors

in my home

Administering medications that have

been discontinued

Other type of error (please give examples) _____________________________________________

**7. Which do you think are the most common errors of medication accountability? Please rate from 1 – 7 with 1 being is the ‘most common’ and 7 being the ‘least common’. (*Interview question: which if any of these errors have you seen in your home?)***

Not signing for medication given

Not recording reasons for non-administration

Not recording actual amount given for variable dose prescriptions (e.g. “1 or 2 to be given”)

Not recording time given for PRN medications

Not booking in supplies

Not having a witness sign for changed made to the MAR

Other type of error (please specify)______________________________________

I have not seen any of these errors

**8. How long ago did you last attend drug administration training?–––––––––––––months/years**

**9. Did your training involve looking at the side effects of common medications?**

Yes  No

**10. Did your training involve looking at what some common medications do?**

Yes  No

**11. Do you know the purpose for of all of the drugs that you give out?**

Always  Almost always  Sometimes  Almost never  Never

**12. In a normal week, how often would you administer drugs?_______________times per/week**

**13. How confident are you that your current drug administration system is:**

**(a) Safe: residents get correct medication at correct time (Please tick the answer that best matches how you feel).**

1. Very confident

2. Fairly confident

3. Neither confident nor lacking confidence

4. Fairly lacking confidence

5. No confidence

**(b) Time efficient re: duration of medication round (Please tick the answer that best matches how you feel).**

1. Very confident

2. Fairly confident

3. Neither confident nor lacking confidence

4. Fairly lacking confidence

5. No confidence

**(c) How confident are you that your current system is the best given the number of staff available to dispense medicines? (Please tick the answer that best matches how you feel).**

1. Very confident

2. Fairly confident

3. Neither confident nor lacking confidence

4. Fairly lacking confidence

5. No confidence

**14. Approximately how long does a drug round take at present? (*Observation: rounds will be observed for shortcuts, adherence to policy, etc. and timed to gather data on actual times before and after PCS introduction this will then be compared to the actual times)***

(a) Early morning _____________________________________ mins____________

(b) Lunchtime _____________________________________mins_____________

(c) Teatime _____________________________________mins_____________

(d) Bedtime ______________________________________mins____________

**15. Are you aware of incidences of any ‘near misses’ (i.e. times where an error has almost occurred but the administrator has noticed just in time) in the home? Yes**  **No**

*(Interview: Please give examples*.) **_________________________________**

**16. Do you generally carry out the drug round: (a)** Alone **or (b)** With another person

**17. How at ease are you with carrying out a drug round on your own? (please circle the number that best fits your level of ease)**

**1 2 3 4 5 6 7**

***Not at all Extremely at ease***

***At ease***

**18. What are the pitfalls/problems associated with your current method of stock control?**

**(please tick any that apply).**

(a) Time consuming

(b) Easy to make a mistake

(c) Run out of stock before next order

(d) Order too much stock

(i.e. potential for stock to go out of date – stock wastage)

(e) Involves too many staff members

(f) Uses too much storage space

(g) No problem

(h) Don’t know how much we have in stock at any one time

**19. If you ticked any of the answers at 16 above, which of these is the most frequent problem and which is the least frequent problem? (please choose a letter from (a) – (g))**

Most frequent problem is_______________________________

Least frequent problem is_______________________________

**Alterations to MARs**

**20. Who is allowed to make changes to MAR sheets (e.g. dosage changes, discontinuation of meds etc)?**

Care Home Manager  Senior RGN  Senior Managers

Care Staff (with med training)  Other RGNs

Other care staff  GP

Other (please state)  ______________________________________

**21. Is a signature required when alterations are made to MARs?** Yes  No

**22. Is a witness signature required when amendments are made to MARs?** Yes  No

**Special Circumstances**

**23. Some meds normally require some form of checking action prior to administration. In your home do you undertake any of the following:**

(a) Pre-issue Pulse recording for digoxin Yes  No

(b) Regular BP monitoring for those on blood pressure medications Yes  No

(c) Glucose monitoring for insulin Yes  No

**If you answered ‘yes’ to any of a, b or c above, please complete questions 22-25. (***Interview: If you answered no to any of these, who is responsible for the giving of these medications? Staff in home, district nurse, self-med etc.)*

**24. Thinking about medications that require some checking action to prior to administration e.g. pulse recording for digoxin, blood glucose monitoring for insulin, etc. have you received training in order to carry out these resident checks?**

*(Interview: If you have NOT received training to carry out these tasks, what happens if a resident is prescribed a drug that requires pulse/BP/blood sugar monitoring before administration****.)***

Yes, I have received training

No, I have NOT received training

**25. If you HAVE received training from whom/where did you receive this training?**

RGN training  In-house training course (i.e. arranged by company)

RGN in my home  GP

District/community nurse  Other (please state)______________________

**26. Where are checks (e.g. pulse/blood sugar recordings) noted?**

On the MAR sheet only  On MAR ***and*** care plan/notes

In residents care/nursing notes plan ***only***  Other (please specify)

_______________

**27. Please read the following statements and answer the questions below:**

***(a) Statement 1: ‘Staff administering medications assume that the content of the blisters is correct and therefore do not need checking thoroughly.’***

Do you think that this statement is true or untrue? True  Untrue

Have you ever come across a situation where the blisters were wrong? Yes  No

Do you think that with Blisters, some people do not make thorough checks: Yes  No

I have found no problems with this Agree  disagree

*(interview question : Can you give me any examples of this?)*

***(b) Statement 2: ‘Staff assume that the blisters on the racks are up-to-date (i.e. no one has taken any off or added any on).’***

Do you think that this statement is true or untrue? True  Untrue

Have you ever come across a situation where the blisters were wrong? Yes  No

Do you think that with Blisters, some people do not make thorough checks: Yes  No

I have found no problems with this Agree  Disagree

*(Interview question : Can you give me any examples of this?)*

***(c) Statement 3: Staff assume that the blisters on the racks are placed in the correct residents section.***

*(Interview question : Can you give me any examples of this?)*

Do you think that this statement is true or untrue? True  Untrue

Have you ever come across a situation where the blisters were wrong? Yes  No

Do you think that with Blisters, some people do not make thorough checks: Yes  No

I have found no problems with this Agree  Disagree

***(d) Statement 4:* *Interim medicines can be supplied in the middle of the month. Because they are supplied in the middle, blisters may not be placed on the racks in the correct position. Thus, there is a risk of them getting missed out of the normal drug administration system.***

*(Interview question: Can you give me any examples of this?)*

Is this a real risk? Yes  No

Have you ever seen medicines being missed under this circumstance? Yes  No

Have you ever come across blisters placed on the racks in the incorrect position Yes  No

***(e) Statement 5: ‘Interim medicines, can be supplied in the middle of the month. Because they are supplied in the middle, there is a risk of some medications that are not blistered (because they are not able to go on the racks e.g. may be in the fridge) being missed.’***

*(Interview question: Can you give me any examples of this?)*

Is this a real risk? Yes  No

Have you ever seen medicines being missed under this circumstance? Yes  No

***(f) Statement 6: ‘Because everything is supplied in blisters, dose changes during the month would have to be added to, or removed from the racks, thus there is risk of medicines not being administered properly’.***

Do you think that this statement is true or untrue for your home? True  Untrue  *(Interview: Prompt if busy, if forgetful, if new member of staff)*

Have you ever come across situations where the changes were not made? Yes  No

Are there other risks? Yes  No

(*Explore in interview if yes)*

***(g) Statement 7: ‘The racking system presents some difficulties.’***

Do you think that this statement is true or untrue for your home? True  Untrue

*(If true explore through interview)*

Is the system bulky? Yes  No

Is it a pain to have to swap the different racks round? Yes  No

Do you think it is easy to pop out the tablets from the racks? Yes  No

Do you ever find that the blisters are not on the right racks? Yes  No

Do you ever find that the blisters are not in the right order? Yes  No

Do you ever find someone’s blisters in the wrong section of the rack? Yes  No

Does opening blisters ever injure your fingers? Yes  No

I do not find any problems with the racking system Agree  Disagree

***(h) Statement 8: ‘Blisters are on the racks in the order that the patients usually have their medicines, but sometimes residents are not there when it is their turn and could risk getting missed.’***

Do you think that this statement is true or untrue for your home? True  Untrue

Have you ever known it to happen? Yes  No

What method is used to prevent this happening? (Please tick all that apply)

MAR  Check Blisters at end of round  Notepad  No prompt required

Other (please specify)

***(i) Statement 9: MAR charts are easier to use when additional identifiers are used (colour coding or other similar) to show you which medicines are due and at which time.***

Is this statement true or untrue? True  Untrue

*Interview: If true what types of identifiers are used and what difficulties do these present*

Do you think there is a greater risk of medicines being missed when MAR charts do not have additional identifiers e.g. colour coding? Yes  No

Have you ever come across an instance when the colour coding was wrong? Yes  No

(*interview: please give examples if answered yes)*

*(interview: Who is responsible for colour coding? Is it always the same person/different person?*

***(j)* *Statement 10: One thing that CSCI inspectors look for on MAR sheets is missing entries.***

Were you aware of this? Yes  No

Why do you think missing entries are not recorded?

Time pressure  Not enough space on MAR charts  I have found no Problem with this

Other reasons (please specify)

***(k)* *Statement 11: ‘CSCI inspectors also look for recorded reasons why medications have not been given.’***

Were you aware of this? Yes  No

Why do you think reasons for non-administration are not recorded?

Time pressure  Not enough space on MAR charts  I have found no Problem with this

Other reasons (please specify)

______________________________________________________________________________

***(l)* *Statement 12: ‘CSCI inspectors look to see whether the number/dose of PRN medication is recorded on the MAR sheets.’***

Were you aware of this? Yes  No

Why do you think the number/dosage of PRN medications is sometimes not recorded?

Time pressure  Not enough space on MAR charts

I have found no Problem with this  Other reasons (please specify)

______________________________________________________________________________

***(m) Statement 13: ‘Sharing of some resident medicines, e.g. Lactulose and Movicol is unavoidable.’***

Do you think that this statement is true or untrue for your home? True  Untrue

Why do you think this happens? (please tick any that apply)

Not enough room on trolley  Residents own stock has run out

New medication has been prescribed, so no stock available for that resident

Other (please state) ______________________________________________________________

Have you ever seen this practice of sharing?

Frequently  Fairly frequently  Rarely  Never

Was it to do with storing and finding the medicines within the trolley Yes  No

Do you think being able to store this type of medication within the trolley,

would reduce the incidence of sharing medicines? Yes  No

***(n) Statement 14: ‘New entries indicating any medication changes are usually made as new entries and countersigned.’***

Do you think that this statement is true or untrue for your home? True  Untrue

Do you come across occasions where the MAR chart has been changed rather

than a new entry made? Yes  No

Do you come across occasions where the changes are not signed by two people? Yes  No

Do you sometimes find it difficult to decipher other people’s handwriting? Yes  No

***(o) Statement 15: ‘Some residents may have a number of MAR sheets plus an interim MAR sheet which may be placed at the back of existing sheets. This increases the risk of medications being missed.’***

Do you think that this statement is true or untrue for your home? True  Untrue

Have you seen this happen?

Frequently  Fairly frequently  Rarely  Never

**28. When you are off for a few days how do you inform yourself of medication changes? Please tick any that apply.**

Study MAR charts  Discuss with colleagues  Ask residents  Other (Please specify)

________________________________________________________________________________

**29. When do you usually sign the MAR sheets**

Sign before potting  sign after potting  Use both practices equally

Other (please specify) __________________________________________________

Have you ever seen or suspected that MAR charts have been signed on mass?

Yes  No

*(Explore at interview: Why sign when you sign? How do you know which medicines have been potted if you’re interrupted? How do you ensure that you remember to go back and sign the MAR chart, if you were interrupted?)*

**30. What is your opinion of the MAR chart folder?**

I have no problems with the MAR chart folder Yes  No

Do you find it too bulky? Yes  No

Is it easy to find patients MAR charts in the folder? Yes  No

Do the MAR chart holes get damaged and slide out? Yes  No

Other (please state)_____________________________________________________

**31. What is your attitude towards the introduction of a new medication system to replace the one you are using?**

Very keen  Fairly keen  Neither keen nor reluctant  Fairly reluctant

Very reluctant

**32. Of the following, who do you think holds a positive attitude towards changing to a new medication system? (Tick all that apply)**

Care Home Manager  Senior RGN  Senior Managers

Care Staff  Other RGNs  Residents/Relatives

Senior Care staff  GP

Other  (please specify) ______________________________________

*Who is the most positive and who is the least positive?__________________________*

**33. Who of the following do you think holds a negative attitude towards changing to a new medication system? (tick all that apply)**

Care Home Manager  Senior RGN  Senior Managers

Care Staff  Other RGNs  Residents/Relatives

Senior Care staff  GP

Other  (please specify) ______________________________________

*Who is the most negative and who is the least negative?__________________________*

**Computer use**

This section will ask you a series of questions regarding your use of computers in the home and at work.

**34. How often do you use a computer at home? (Please tick the most appropriate answer)**

Never  Daily  Weekly  Monthly

**35. What do you use a home computer for? (Please tick all that apply)**

Playing games  Spreadsheets

Word processing  Email

Internet for information gathering  Internet for finance

Internet for chat/discussion rooms  Internet for shopping

Other (please give details)___________________________________________________

**36. How often do you use a computer at work? (please tick the most appropriate answer)**

Never  Daily  Weekly  Monthly

**37. What do you use a work computer for? (Please circle all that apply)**

Patient data/records (e.g. blood results, x-rays, etc)  Email

Ordering/stock control  Word processing

Management (e.g. off duty, bed status)  Playing games

Internet for information gathering  Spreadsheets

Internet for chat/discussion rooms  Internet for shopping

Other (please give details) _______________________________

**38. Do you have any formal training in computer use (e.g. CLAIT, RSA, ECDL)? (Please**

**circle the appropriate answer)**

Yes  No

**39. How would you rate your experience in terms of computer use? (Please circle the**

**appropriate answer)**

Inexperienced 0 1 2 3 4 5 6 7 8 9 10 Experienced

**40. How would you rate your confidence in terms of computer use? (Please circle the**

**appropriate answer)**

Confident 0 1 2 3 4 5 6 7 8 9 10 Low confidence

**Mobile Phones**

The following section asks you about your mobile phone use.

**41. Do you own a mobile phone? Yes**  **No**

**42. Do you own a PDA or smartphone? (E.g. iPhone, Blackberry, Palm etc) Yes**  **No**

**43. What kind of things do you regularly do with your mobile phone/smartphone? (Tick all that apply)**

Make calls  Text people

Listen to music  Take photographs

Check emails  Surf the internet

Create documents  Instant messaging

Play games  Other (please specify) ________________________

**The Pro-active Care System and Medicines Management in Care Homes: An Exploratory Study of its Impact**

**Pre-PCS Introduction SWOT ANALYSIS; Think about your current system of medication ordering, supply, storage, administration before answering the following questions 44-47**

**44. Please identify up to 5 strengths for your current system of medication ordering, supply, storage and administration. Explain briefly why for each, then rate them on a scale of 1-5, with *1 = unimportant, 2 = fairly unimportant 3 = neither unimportant nor important, 4 = fairly important 5 = very important***

| **Strengths** | **Why?** | **Rate** |
| --- | --- | --- |
|  |  |  |
|  |  |  |
|  |  |  |
|  |  |  |
|  |  |  |

**45. Please identify up to 5 weaknesses for your current system of medication ordering, supply, storage and administration. Explain briefly why for each, then rate them on a scale of 1-5, with *1 = unimportant, 2 = fairly unimportant 3 = neither unimportant nor important, 4 = fairly important 5 = very important***

| **Weaknesses** | **Why?** | **Rate** |
| --- | --- | --- |
|  |  |  |
|  |  |  |
|  |  |  |
|  |  |  |
|  |  |  |

**46. Please identify up to 5 opportunities to strengthen your current system of medication ordering, supply, storage and administration. Explain briefly why for each, then rate them on a scale of 1-5, with *1 = unimportant, 2 = fairly unimportant 3 = neither unimportant nor important, 4 = fairly important 5 = very important***

| **Opportunities** | **Why?** | **Rate** |
| --- | --- | --- |
|  |  |  |
|  |  |  |
|  |  |  |
|  |  |  |
|  |  |  |

**47. Please identify up to 5 threats to your current system of medication ordering, supply, storage and administration. Explain briefly why for each, then rate them on a scale of 1-5, with *1 = unimportant, 2 = fairly unimportant 3 = neither unimportant nor important, 4 = fairly important 5 = very important***

| **Threats** | **Why?** | **Rate** |
| --- | --- | --- |
|  |  |  |
|  |  |  |
|  |  |  |
|  |  |  |
|  |  |  |

**Sources of Job Pressure**

**The following section asks you questions about the pressures that you may come across as part of your work.**

**Using one of the following numbers, please indicate against each of the following items A-Z, your level of job pressure.**

**1 = no pressure**

**2 = slight pressure**

**3 = moderate pressure**

**4 = considerable pressure**

**5 = high pressure**

***Number***

1. **Increased demands from residents. __________**
2. **Inappropriate demands from residents. __________**
3. **Dealing with problem residents. __________**
4. **Dealing with very ill residents and their relatives. __________**
5. **Dealing with earlier discharges from hospital. __________**
6. **Worry about complaints/litigation. __________**
7. **24-hour responsibility for residents. __________**
8. **Working environment and home set-up. __________**
9. **Insufficient time to do justice to the job. __________**
10. **Fear of assault at work. __________**
11. **Disturbance of home/family life by work. __________**
12. **Dividing time between work and spouse/family. __________**
13. **Unsociable hours. __________**
14. **Unrealistic high expectations of role by others. __________**
15. **Insufficient resources within the home. __________**
16. **Dealing with conflict within the home. __________**
17. **Long working hours. __________**
18. **Paperwork. __________**
19. **Organisational changes in the homes __________**
20. **Adverse publicity by media. __________**
21. **Lack of support within home. __________**
22. **Emphasis on resource issues in the home. __________**
23. **The pace of change within homes. __________**
24. **Professional isolation. __________**
25. **Increased workloads. __________**

**Z. Lack of appreciation from residents. __________**
