# Supplementary material for: Medication administration errors for older people in long-term residential care
Source: BMC Geriatr. 2011 Dec 7;11:82. doi: 10.1186/1471-2318-11-82 (PMC3254134; doi:10.1186/1471-2318-11-82)
Supplement: Additional file 3 — Comparison of current research and Barber et al. study (2009). This file provides a comparison of the present research and the only other large scale study of medication administration errors in UK care homes, including details of: • study sites. • site selection process. • resident sample studied. • medication administrations observed. • administration errors recorded. [file 1471-2318-11-82-S3.DOC]

# Additional files

### Additional file 3 – Comparison of current research and Barber *et al*. study (2009)

| **Characteristic** | **Barber *et al [17]*** | **Current study** |
| --- | --- | --- |
| Study sites | - 55 care homes  - Breakdown: 5 nursing, 12 residential, 38  residential & nursing homes | - 13 care homes  - Breakdown: 4 nursing, 9 residential homes |
| Site selection process | - Homes selected via Bradford, London,  Cambridgeshire PCTs  - Large and small chains; single owner;  voluntary sector; LA homes.  - Size 20-29 places most common | - Homes selected based on technology (PCS **^1^**)  Availability in North West, South West, South.  - Large and small chains; single owner;  independent sector homes.  - Size 25-68 places (30-50 places most common) |
| Resident sample studied | - 256 residents sampled  - Random sample  - 256/ 399 (total residents approached)  - Breakdown: 117 nursing, 139 residential  - Mean number medications/ resident = 8.0 | - 345 residents included  - All residents in receipt of medication  - 345/ 463 (total resident places)  - Breakdown: 91 nursing, 254 residential  - Mean number medications/ resident = 8.8 |
| Medication administrations observed | Information collected by observing medication rounds and using MARS **^2^**:  - observation of two medication rounds per  resident  - morning & tea-time rounds only  - 1 or 2 (max) days data on each resident  - total 512 resident medication rounds. | Information collected by downloading data from system:  - data collected on ~336 medication rounds per  resident.  - morning, lunch-time, tea-time & night time rounds  - 84 days data on each resident  - total 188,249 medication administrations |
| Administration errors recorded | *If found that:*  - medication incorrect  - un-prescribed medication  - timing error if has clinical significance  - extra dose  - wrong dose **^3^**  - omissions **^3^**  - other: deteriorated medication, different formula, route error **^3^** | *If recorded attempt to:*  - give medication to wrong person  - give discontinued medication  - give medication at wrong time (including too early, already given, wrong day)  - N/A  - N/A  - N/A |

**^1^** PCS = Proactive Care System which includes pharmacy-managed barcode medication administration system

**^2^** MARS = Medication Administration Recording System (paper-based)

**^3^** PBAS technology does not allow this type of medication administration error
